# Biomedical engineer’s guide to the clinical aspects of intensive care mechanical ventilation

**DOI:** 10.1186/s12938-018-0599-9

**Published:** 2018-11-12

**Authors:** Vincent J. Major, Yeong Shiong Chiew, Geoffrey M. Shaw, J. Geoffrey Chase

**Affiliations:** 1 Department of Population Health, NYU Langone Health, New York, NY USA; 2 School of Engineering, Monash University Malaysia, Subang Jaya, Malaysia; 3 Department of Intensive Care, Christchurch Hospital, Christchurch, New Zealand; 4 Centre for Bioengineering, University of Canterbury, Christchurch, New Zealand

**Keywords:** Mechanical ventilation, Intensive care, Respiratory failure, Model-based treatment, Patient-specific ventilation, Protective lung strategies

## Abstract

**Background:**

Mechanical ventilation is an essential therapy to support critically ill respiratory failure patients. Current standards of care consist of generalised approaches, such as the use of positive end expiratory pressure to inspired oxygen fraction (PEEP–FiO_2_) tables, which fail to account for the inter- and intra-patient variability between and within patients. The benefits of higher or lower tidal volume, PEEP, and other settings are highly debated and no consensus has been reached. Moreover, clinicians implicitly account for patient-specific factors such as disease condition and progression as they manually titrate ventilator settings. Hence, care is highly variable and potentially often non-optimal. These conditions create a situation that could benefit greatly from an engineered approach. The overall goal is a review of ventilation that is accessible to both clinicians and engineers, to bridge the divide between the two fields and enable collaboration to improve patient care and outcomes. This review does not take the form of a typical systematic review. Instead, it defines the standard terminology and introduces key clinical and biomedical measurements before introducing the key clinical studies and their influence in clinical practice which in turn flows into the needs and requirements around how biomedical engineering research can play a role in improving care. Given the significant clinical research to date and its impact on this complex area of care, this review thus provides a tutorial introduction around the review of the state of the art relevant to a biomedical engineering perspective.

**Discussion:**

This review presents the significant clinical aspects and variables of ventilation management, the potential risks associated with suboptimal ventilation management, and a review of the major recent attempts to improve ventilation in the context of these variables. The unique aspect of this review is a focus on these key elements relevant to engineering new approaches. In particular, the need for ventilation strategies which consider, and directly account for, the significant differences in patient condition, disease etiology, and progression within patients is demonstrated with the subsequent requirement for optimal ventilation strategies to titrate for patient- and time-specific conditions.

**Conclusion:**

Engineered, protective lung strategies that can directly account for and manage inter- and intra-patient variability thus offer great potential to improve both individual care, as well as cohort clinical outcomes.

## Background

In intensive care, mechanical ventilation (MV) is the primary support for patients with respiratory failure or acute respiratory distress syndrome (ARDS) despite decades of research [1]. MV is an essential life support, but if non-optimally managed, it can also degrade patient condition [2, 3]. MV provides positive airway pressure and airflow to support work of breathing, sustain oxygenation and enable patient recovery. In particular, the goal of MV is to provide these supports while protecting the lung from further damage [1, 4]. However, while there is some general agreement on which MV settings and clinical parameters are preferred [1, 5, 6], there are limited guidelines and conflicting trial results [7–13] in optimising MV support, resulting in variable MV settings. (Please refer to these recent reviews of clinical advances in treatment [1] and challenges [14] of MV for ARDS.) Moreover, MV care and guidelines are almost entirely clinically determined in contrast to the area of diabetes/metabolism, for example, which has a long history of mathematical and engineering research that has driven recent advances in care.

An example of highly variable MV settings is the positive end-expiratory pressure (PEEP). Some may consider lower PEEP superior and safer [15, 16]. However, lower PEEP can lead to increased cases of oxygen desaturation and hypoxemia [8, 17] and atelectrauma, indicated by a greater number of rescue therapies and death after rescue therapy [13]. In contrast, higher PEEP can increase recruitment of collapsed lung units [18–20], stabilising injured or collapsed alveoli [21, 22], reducing inflammatory mediators in plasma and bronchoalveolar lavage fluid [23]. However, higher PEEP can also cause ventilator induced lung injury (VILI) [8, 13]. Hence, there is no consensus on the best PEEP, which can vary between and within patients.

Optimal settings are patient-specific and likely evolve over time, and are thus not easily titrated using standard clinical protocols. Because current tools and methods cannot provide enough insight into patient-specific response to MV in real-time [24], particularly breath-to-breath or hour-to-hour, and non-invasively, the best approach in setting MV remains uncertain [3, 13, 21, 22, 24–27]. As a result, the current standard of MV therapy in the intensive care unit (ICU) relies heavily on clinician experience and intuition, or a generalised one size fits all approach, such as setting MV for acute respiratory distress syndrome (ARDS) patients using the ARDSNet or lung protective strategies recommendations [5, 8, 10, 11]. Therefore, patient-specific MV methods are needed to improve individual patient outcomes beyond where they are today [24].

More specifically, the use of biomedical devices and model-based methods enable broader and wider perspective on patient-specific condition in setting MV [24]. However, research in this area is still lacking. Of particular concern, is that MV is a highly integrated field, mixing medical knowledge with engineering technologies, including control systems, signal processing and mechatronics. As such, technologists and engineers rely heavily on clinical expertise for guidance, potentially without proper understanding of the problem, creating a need to bridge this lack of medical knowledge.

This paper addresses this issue via a comprehensive review of the clinical aspects and application of MV for readers of both clinical and non-clinical expertise. A fundamental introduction to MV is included first to define the clinical terminology used throughout and to organize the review, which, despite being medically focused, aims to maximize comprehension by non-clinical readers and offer entry points from the engineering perspective to understand the fundamental mechanics at play. In particular, the variables and mechanics of MV are placed in a structure that is also suitable for engineering mechanics analysis, with relevant clinical references on their use.

The article covers the adverse effects of suboptimal MV settings, before discussing several large clinical randomised controlled trials concerning different MV strategies and recent trends, all of which set the clinical context and better define the clinical shortfalls that exist today. The MV strategies presented are classified into families based on how they intend to mitigate the challenges of MV. This is a new method of assessing the field and translating engineering solutions to medical problems, and vice versa. The overall goal is to delineate the clinical state of the art to highlight the need for patient-specific methods in standardising MV settings, ending with a particular focus on how novel engineering, modelling and simulation approaches to measurement, control and/or management could have significant clinical impact. It thus provides a clinical state of the art tutorial review to motivate potential avenues for future engineering science research to transform this ubiquitous critical care therapy.

Hence, the overall goal of this review is to provide a broad overview, particularly for non-specialists or those new to this area so they can rapidly assimilate the state of the art, rather than a detailed, systematic review of a particular facet, clinical or engineering. Thus, in this case, the review covers both clinical definitions, to introduce terminology and its clinical and engineering meaning, as well as to discuss the clinical state of the art, and further provide context for the mechanics and biomedical engineering aspects. As a result, some areas may appear overly simplified, to those familiar with that portion of the problem, but are necessary for those who are seeking a foothold. Throughout, we will attempt to point the reader to recent, more specific, reviews that encompass the greater research area.

## Fundamentals of mechanical ventilation

### Mechanical ventilation parameters

Key parameters of MV supported breathing cycles include: tidal volume, airway pressure, peak inspiratory pressure, plateau pressure, positive end-expiratory pressure, fraction of inspired oxygen, respiratory rate and inspiration to expiration ratio.

#### Tidal volume (V_*t*_)

Physically, V_t_ is the air volume entering and exiting the lungs each breath. V_t_ is chosen by the clinician, usually using predicted body weight, and for ARDS patients V_t_ between 4 and 8 mL/kg is recommended [28]. Higher tidal volume can assist with removal of carbon dioxide from the lung in patients with hypercapnia or delivery of oxygen to patients that have hypoxemia. However, excessive volumes can also overinflate and stretch lung tissue causing injury [29, 30].

#### Fraction of inspired oxygen (FiO2)

FiO_2_ is the oxygen concentration delivered to the patient. Higher FiO_2_ enables better exchange oxygen from the lungs into the blood, increasing the partial pressure of oxygen in the alveoli and thus the rate of diffusion. FiO_2_ above 21% (atmospheric air) is often used to increase oxygenation, while avoiding the risks associated with delivering higher pressures and tidal volumes, or when collapsed alveoli are not recruitable [31]. However, excessive O_2_ partial pressures can cause oxygen toxicity [32, 33].

#### Airway pressure (P_*aw*_)

The *P*_*aw*_ is the pressure supplied from the ventilator to the patient during MV. There are four distinct measures of *P*_*aw*_ during a typical MV breathing cycle:

- Positive end expiratory pressure (PEEP): PEEP is the elevated airway pressure at the end of expiration. PEEP is an important setting used to maintain lung recruitment to allow gas-exchange [34, 35]. It also prevents the cyclic opening and closing of collapsed lung units (atelectasis), which can cause further damage [2, 30]. Titrating PEEP is often a topic of debate, with some advocating higher levels, and some lower levels [8]. It is currently most often set using the PEEP–FiO2 table [5].
- Peak inspiratory pressure (PIP): PIP is the maximum airway pressure during inspiration. PIP is limited to avoid excessive pressures causing further injury [2, 36, 37]. PIP may be limited in pressure control modes where the pressure range can be set.
- Plateau pressure (*P*_*plat*_): Plateau pressure is the airway pressure measured during an end of inspiratory pause [38]. Compared to PIP, this pressure level is lower, as it is not influenced by the dynamic pressure differences due airway resistance. This pressure level is used as a representation of the pressure in the alveoli, and is often used as a threshold for high-pressure levels. Typically, airway pressure is set at pressure level where *P*_*plat*_ is less than 30–35 cmH_2_O to avoid barotrauma [39].
- Driving pressure (*ΔP*): ΔP is the pressure difference added above PEEP to *P*_*plat*_. Recent post hoc and meta-analyses have suggested that driving pressure may be more important than other MV parameters determining outcomes [40, 41] that higher driving pressures are associated with increased mortality [42]. Crucially, these results and, any new guidelines, must consider ΔP with respect to at least one of PEEP and *P*_*plat*_.

**Fig. 1:**
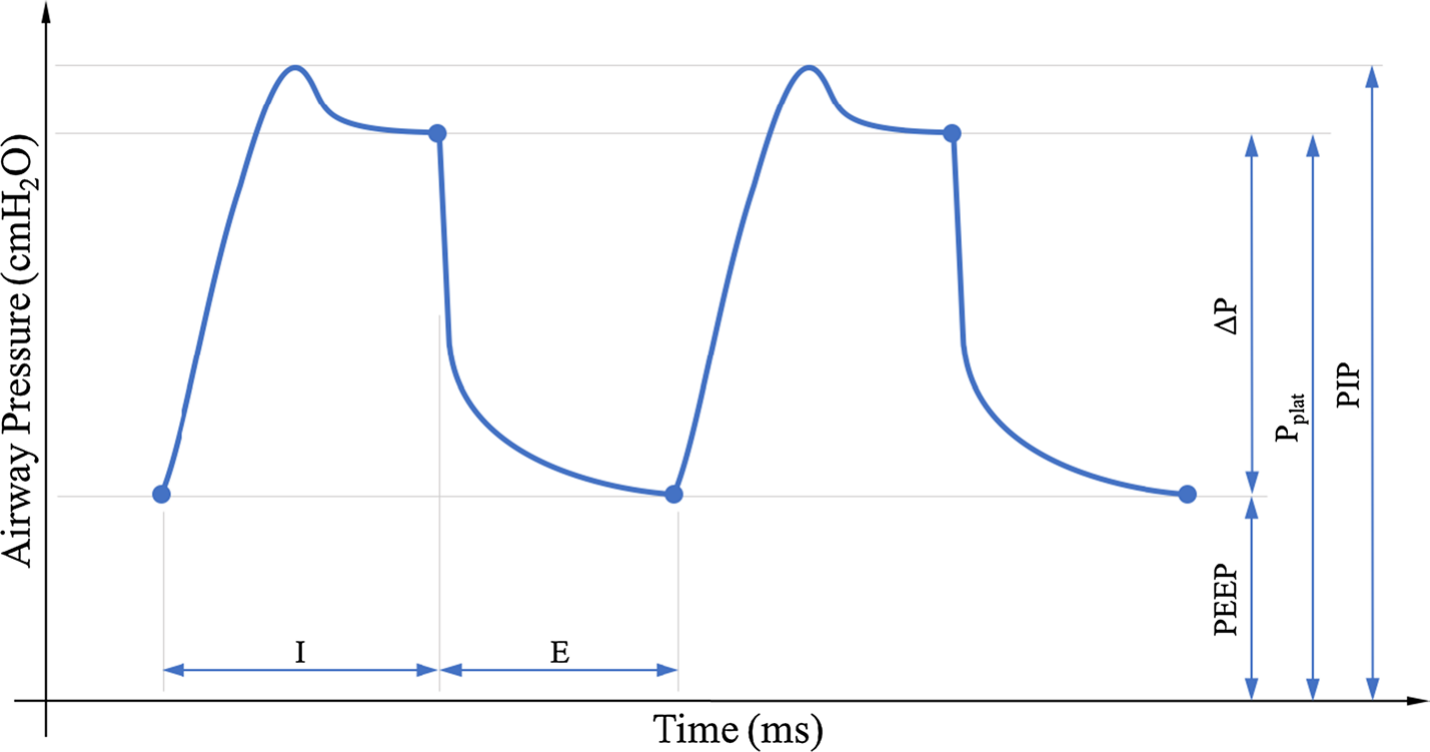
An idealized MV breath cycle highlighting common pressure measurements including positive end expiratory pressure (PEEP), driving pressure (ΔP), peak inspiratory pressure (PIP), and plateau pressure (P_*plat*_) and the two time components, inspiration (I) and expiration (E), that determine the I:E ratio. These measures are the most commonly monitored in practice and employed in modelling and moreover as a set can roughly describe both inspiration and expiration

The four primary *P*_*aw*_ measures do not completely define a breath cycle but, as described in Fig. 1, do measure the extremes of both inspiration and expiration. Together with the time components, this set of metrics can roughly describe the entire breathing cycle with only minimal redundancy.

#### Respiratory rate (RR) and inspiration to expiration (I:E) ratio

The RR during MV is the number of breaths per minute. It is commonly around 16–20 so that each breath is approximately 3–4 s in length. During fully controlled ventilation, within the length of each breath (corresponding to the RR), clinicians can adjust the I:E ratio—the ratio of inspiration time to expiration time—to ensure adequate ventilation of carbon dioxide out of the lung. Setting RR together with *Vt* also ensure adequate minute ventilation (litres of air per minute). Spontaneously breathing patients on support modes set RR themselves, and hybrid modes, such as mandatory ventilation, ensure a minimum allowable RR is met.

### Ventilation modes

**Fig. 2:**
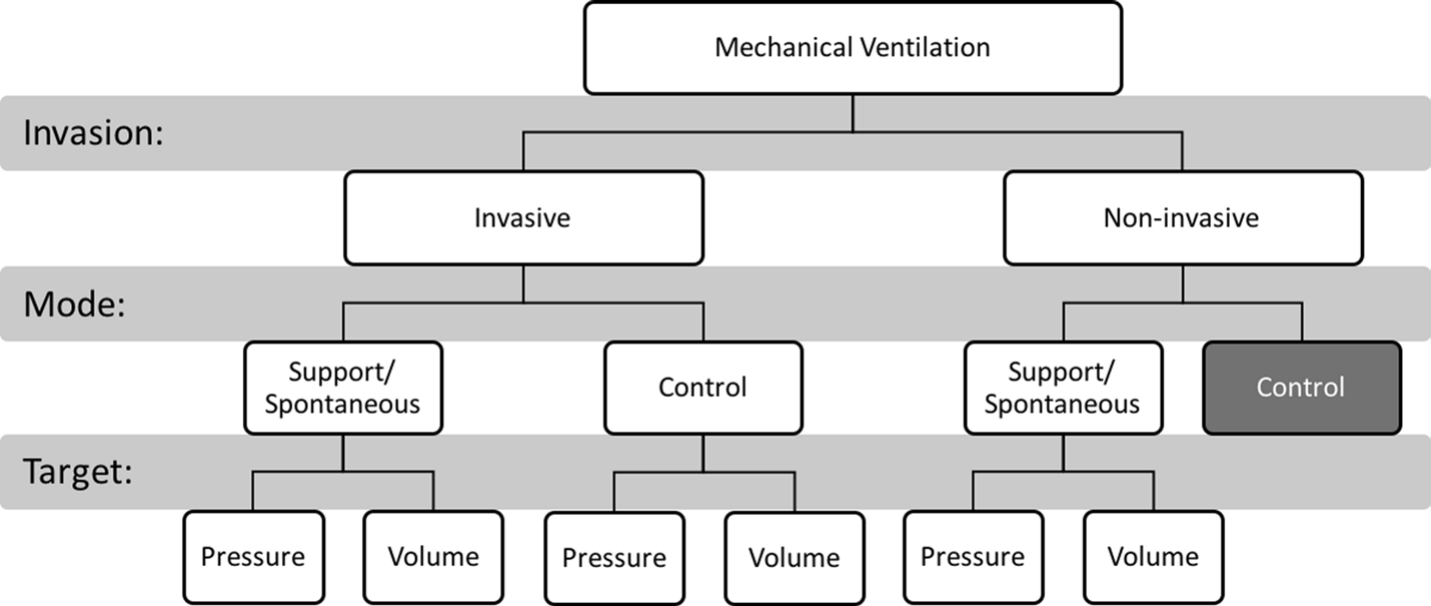
A simple schematic diagram describing how MV is specified by selecting one option from each of three classes. Typically, the type (invasive vs non-invasive) is selected first, then the mode (support/spontaneous vs control), and finally the target (pressure vs volume). These three dimensions partition the possible combinations into six commonly employed sets (omitting non-invasive control ventilation)

Fundamentally, ventilation can be divided into several groups by the level of invasion, the mode used and the target. Typically, MV is first split into invasive or non-invasive, determined if the patient is intubated or not, and then subdivided into control or support modes, depending on the patient’s breathing efforts and sedation, and finally, pressure or volume controlled modes [43–45]. Figure 2 describes three tiers that roughly align with the decision-making process to completely define each of the three dimensions. The box representing control mode non-invasive ventilation is shaded to designate that this combination of settings is rarely employed [45]. Please refer to a recent review of ventilation modes and settings in the context of non-invasive ventilation [45].

1. Invasive versus non-invasive:
  MV can be delivered invasive or non-invasively. Invasive ventilation involves insertion of an endotracheal tube or tracheotomy, whereas non-invasive ventilation (NIV) is delivered via face mask [46]. Invasive ventilation is most common when the ventilator is required to manage the entire patient work of breathing.
  The application of NIV for ARDS remains controversial. Implementation of NIV requires more training of clinical staff [47], remains complicated to correctly identify ARDS patients that are likely to benefit [48], and NIV failure, leading to intubation, is common [49]. In a recent study of 2800 ARDS patients, 15% received NIV and failure rates ranged from 22 to 47% for patients with mild and severe ARDS with hospital mortality rates tripling for patients with NIV failure (45% vs 15% for success) [50]. However, NIV is actively being improved as evidenced by a recent study that terminated early for efficacy. The authors compared outcomes of ARDS patients treated with NIV delivered by helmet, compared to a typical face mask and observed that intubation rates dropped, ventilator free days increased, and 90 day mortality rates decreased [51].
2. Control versus support modes:
  Control ventilation modes strictly adhere to the chosen ventilation strategy, and are commonly used for patients who are heavily sedated, paralyzed, or cannot breathe regularly enough to support themselves. They provide all the work of breathing.
  Support ventilation modes act as an assistant to patients who are breathing spontaneously. Unlike controlled modes, support MV delivers breathing cycles when triggered by patients.
  Currently, hybrid modes, such as synchronized intermittent mandatory ventilation (SIMV), allow patients to control the timing of their breathing, while offering up to full support, but also ensure that mandatory breaths are given to the patient when no spontaneous effort is made. Hybrid modes ameliorate the limitations of set modes.
3. Pressure versus volume control methods:
  Pressure control fundamentally controls the input and output pressure of the ventilation. This mode allows precise limitation of the maximum PIP to prevent barotrauma [52]. During pressure control, the airflow and tidal volume are delivered depending on the controlled pressure.
  Volume control methods enable a set minimal tidal volume during ventilation. In return, the airway pressure is thus determined by the flow and tidal volume profile.
  Similar to control and support modes, hybrids between pressure and volume control ventilation modes have emerged to provide the advantages of both modes [53].

### MV outcome measurements

The quality of MV delivery is often assessed using outcome measurements or surrogates. The following are several outcome measurements often assessed.

*Blood oxygenation* can be measured using arterial blood gas information, partial pressure of oxygen (PaO_2_), or using pulse oximetry, peripheral capillary oxygen saturation (SpO_2_). Blood oxygenation is the most common monitoring metric to ensure ventilation is adequate and delivering sufficient oxygen to the patient. Standard practice aims for minimum PaO_2_ > 80 mmHg [54, 55] and minimum SpO_2_ on the order of 88–95% depending on the current FiO_2_ [8, 10, 11].

*Minute ventilation* is the volume of air delivered during 1 min and is effectively the product of the RR and V_t_ input settings. Target minute ventilation goals are commonly 8–10 L/min for adults [56, 57].

*Transpulmonary pressure* is the pressure difference between the alveoli and the pleural cavity. Airway pressure—the pressure at the airway opening—is easier to measure but may not be as clinically informative as transpulmonary (or esophageal) pressure [58]. One measured airway pressure may not translate into consistent transpulmonary pressures between patients due to the heterogeneity of patient condition [59]. Since the mechanics of lung collapse, recruitment and overdistension depend on the pressure within the lung, transpulmonary pressures are clearly more relevant to the stress and strain applied to the lung and any subsequent injury than measured airway pressure [2, 60]. For this reason, MV should provide adequate transpulmonary pressure to maintain acceptable oxygenation, while minimising both atelectasis and overdistension [2, 27]. However, estimated measures of transpulmonary pressure require the use of a highly invasive balloon catheter to measure the pleural pressure [27] and are thus not often applied clinically. Please refer to a recent review discussing applications of transpulmonary (and esophageal) pressure [58].

Lung imaging techniques create images of the lungs for clinicians to assess lung condition, allowing them to treat the patient based on their observation [24]. *Computed tomography* (CT) is regarded as a gold standard. CT images allow clinicians to assess patient condition, response to PEEP titrations, alveoli recruitment/distension and gas distributions. However, CT for ICU patients is costly, requires transfer to the radiology department or a portable CT machine and further exposes the patient to radiation [24, 61–65]. Hence, it is not a regular care-monitoring tool.

*Portable X*-*rays* remain the most common radiographic examination [64, 65] as they can be carried out at the bedside and subject the patient to a smaller radiation dose. However, portable radiographs have lower and more variable quality [63, 65] and commonly only show one two-dimensional, frontal plane, compared to images in multiple planes with varying slice thickness available in CT, helical CT or other three-dimensional techniques [64, 65]. Finally, and most importantly, imaging methods that use ionising radiation are not suitable for continuous or semi-continuous monitoring of lung condition. Thus, the application of such imaging techniques to guide therapy remains limited [66].

An emerging form of lung imaging is *electrical impedance tomography* (EIT). EIT has shown good correlation with CT, such that it has been proposed and recently validated to guide ventilation therapy [67–69]. Commercial EIT systems generate lung images using relative impedance changes with respect to end-expiration, allowing ventilation recruitment and inhomogeneity to be monitored in real-time [68, 70]. Several recent studies have demonstrated the utility of EIT at the bedside by titrating PEEP to ensure protective lung strategies balance recruitment without overdistension [71–73]. Moreover, EIT has shown good correlation with CT [74] but is not yet ready to replace CT as a gold standard as the technology is relatively new and costly [75]. A recent review described EIT as clinically validated but noted the literature lacks evidence to its efficacy in clinical outcomes [69]. More studies are thus required for this technology to implement EIT as part of regular patient care. Please refer to these recent reviews for further reading in the clinical [69, 70] and bioengineering [76] state of the art.

Other examples of outcome measurements that can quantify the quality of care for mechanically ventilated patients in the ICU include:

- Length of mechanical ventilation (LoMV) and/or ventilation free days (VFD): LoMV is quantified as how long the patient requires mechanical ventilation support and VFD is measured as the number of days free from ventilator support within a 28-day period.
- Mortality: Measured as survived or deceased within a time-period. Common examples are ICU mortality, hospital mortality, 28-day mortality, and 90-day mortality,
- Severity scoring systems: Patient severity scores and metrics are used to account for the severity of the patient’s disease state. Several common examples are the Acute Physiology and Chronic Health Evaluation score (APACHE) [77–79], Simplified Acute Physiology Score (SAPS) [80, 81], and Sequential Organ Failure Assessment score (SOFA) [82].

### Summary

ICU MV treatment is managed by setting several basic ventilation parameters, while checking that other measured parameters are within acceptable bounds. This process is further complicated while intermittently making small changes to care based on patient response. It is common to titrate the ventilation parameters manually to ensure all targets are met, while trying to reduce the risk of further injury. The lack of clear consensus combined with the wealth of MV modes and settings ensures significant inter- and intra-patient variability in care.

## The problem with MV management

### Ventilation induced lung injury (VILI)

MV is a crucial support for patients with respiratory failure. However, this essential treatment can have (unintended) harmful consequences, causing further injury and/or delay to recovery. Ventilator induced lung injury (VILI) is caused by non-optimal MV and manifests as a mechanical injury to alveoli that exacerbates the systemic inflammation [23, 30, 70, 83–91]. It can thus directly increase the risk of death [88], as well as length and cost of ventilation treatment [92].

A normal, healthy person creates a negative-pressure inside the lung by expanding the chest wall and contracting the diaphragm, which creates a negative pressure in the pleural cavity, and in the lung itself. When the pressure inside the lung is lower than ambient, air flows into the lung. During expiration, air is exhaled passively, reducing the volume of the lung. Modern MV is comprised of positive-pressure ventilation that drives air and oxygen into the lung during inspiration and lets expiration occur passively. This non-physiological inspiration during MV support, may add stress and strain to lung tissues, resulting in trauma in any of four categories: barotrauma, volutrauma, atelectrauma, and biotrauma or other [2, 93, 94]. For further discussion on the pathophysiology of VILI and several approaches to minimize VILI, please refer to a review by Fan et al. [95].

*Barotrauma* is injury caused by excessive pressures in the lung. The pressure gradient between the alveoli and the abdomen can cause air to migrate into the interstitial tissue causing many of the manifestations of barotrauma [2, 29]. Early work by Peterson et al. [36] reported that all patients with PEEP > 40 and/or PIP > 100 cmH_2_O developed barotrauma. However, more recently, Weg et al. [96] cast doubt on these results when they compared patients with matching disease states (ARDS induced by sepsis) and reported no significant difference in pneumothorax rates for high pressures or volumes. Thus, the pressure at incidence is likely patient-specific [97]. Overall, barotrauma is a result of driving pressure and/or PEEP that is too high, resulting in excessive PIP and lung pressures.

*Volutrauma* occurs when ventilation with excessive volume stretches the lung tissue beyond its elastic limit causing injury. It can lead to pulmonary edema, increased fluid filtration, diffuse damage to alveoli, epithelial and microvascular permeability [2, 29]. It is a result of too large tidal volume either by specification or as a result of delivered driving pressure in pressure controlled modes resulting in too much volume expansion.

*Atelectrauma* is a lung injury that occurs when ventilation with too little volume and/or pressure causes repeated, cyclic opening and closing of unstable alveoli near the boundary of collapsed and aerated areas [1, 94, 98]. MV uses PEEP and higher airway pressures to recruit alveoli during inspiration. As the pressure drops during expiration, some diseased alveoli collapse and reopen in the next breath. This cyclic opening and closing is a symptom of a PEEP that may be too low to keep such recruited alveoli open. Atelectrauma is common in patients diagnosed with ARDS and may be more significant than originally thought [10]. Hence, patient-specific pressure and PEEP is critical.

*Biotrauma* is lung injury caused by the body’s response to the invasion of MV and is the most difficult lung injury to quantify in a clinical setting. MV can cause increases in alveolar-capillary permeability, surfactant inactivation and the release of inflammatory mediators [99–101]. Slutsky et al. [102] and Murphy et al. [103], noted that it can lead to multiple organ failure. Non-protective ventilation strategies (e.g. V_t_ ~ 12 mL/kg and PEEP = 0 cmH_2_O) are associated with bacterial translocation and the transmission of pulmonary infections and inflammatory mediators into the circulatory system with subsequent systemic inflammation, compared to protective ventilation strategies with moderate PEEP (10–12.5 cmH_2_O) and lower tidal volume ventilation (~ 5 mL/kg).

Other similar adverse effects of MV include ventilator associated pneumonia [1, 104], pulmonary edema or fluid build-up in the lung [8, 70], circulatory depression or a reduction in cardiac output due to increased chest cavity pressures with PEEP [8], oxygen toxicity due to excessive oxygen from FiO_2_ [1, 2, 101], and hypercapnia or excessive CO_2_ caused by too little removal of CO_2_ out of the blood [1]. All of these effects increase the risk of poor patient outcome.

### Patient intra- inter variability: disease state and variable treatment response

The lung of a respiratory failure patient is very heterogeneous [70], with mixed healthy and diseased alveoli, displaying significant inter- and intra-patient variability. Thus, what works for one patient may lead to VILI in another [21, 105]. Equally, what helps some diseased alveoli, such as added pressure, may injure nearby healthy alveoli. The inability to easily assess lung heterogeneity regularly with imaging, or to selectively provide pressure and volume to areas of the lung thus leads to most of the difficulty in optimising care.

Variability between patients (inter-patient) can be driven by heterogeneous disease and response to treatment [1]. Within a cohort of ARDS or respiratory failure patients, the primary diagnosis is often not recorded as ARDS [106, 107], as the etiology of respiratory failure varies with each patient [108, 109], and ARDS may never be diagnosed, limited by clinical interpretation of chest imaging (please refer to a recent review for discussion surrounding the diagnostic limitations of ARDS [1]). ARDS may be caused by both pulmonary and extra-pulmonary insults [1], which has lead to very diverse ARDS, or respiratory failure, study cohorts [108, 109]. With the diverse causes of respiratory failure comes a wide distribution of lung condition and thus individual patient-specific requirements in optimal ventilation settings.

Disease progression and treatment can affect lung condition and the corresponding optimal ventilation parameters creating variability within one patient (intra-patient) over time. Hence, care must be able to measure appropriate metrics of patient-specific lung condition to evolve over time, as well as trying to ensure care is as optimal as possible at each time point in real-time, rather than intermittently every 6 or 24 h.

Heterogeneity within a patient’s lungs can also lead to variability within one patient (intra-patient) during any one breath. Within the ARDS or respiratory failure lung, it is common for some areas to be collapsed (atelectasis) and poorly perfused, while others are normal. MV cannot provide ventilation separately to the heterogeneous lung areas, though one lung ventilation is possible [110]. Considering both lungs, each with significant variability, selection of ‘optimal’ ventilation settings for overall lung recruitment can provide excessive pressures/volumes to some regions, and too little to others. Lung injury can thus be exacerbated by attempting to provide improved care by further injuring non-aerated, collapsed alveoli. The result is a need to balance, as discussed, the care of injured lung units with the possible harm to unaffected units.

### The influence of the definition and diagnosis of respiratory failure patients

Various pulmonary and extra-pulmonary insults may lead to a patient developing respiratory failure, or the more severe ARDS [1]. The most frequent are pneumonia and extra-pulmonary sepsis [111–113]. The high mortality and morbidity of ARDS patients has made these cohorts a focus of MV research, and optimised MV is critical to outcome [5, 10, 114].

In 1994, the American-European Consensus Conference (AECC) defined acute lung injury (ALI) and ARDS [115] as a syndrome of acute onset of respiratory failure with findings of bilateral infiltrates on chest radiograph. This definition is followed by the absence of elevated left heart filling pressure determined either diagnostically with a pulmonary artery catheter (pulmonary artery occlusion pressure (*PaO*_*2*_) of < 18 mmHg) or clinically (absence of evidence of left arterial hypertension) [115, 116]. A *PaO*_*2*_/*FiO*_*2*_ (P/F) ratio less than 300 mmHg is considered ALI, and *P/F* ratio < 200 mmHg is categorised acute respiratory distress syndrome (ARDS).

This definition was updated in 2012, and is referred to as the ARDS Berlin definition [109]. The changes in definition includes: (1) the replacement of ALI with mild, moderate and severe ARDS; (2) PEEP settings; (3) defining the period of acute onset; (4) recognition of CT imaging as diagnostic; and (5) exclusion of hydrostatic pulmonary edema [117]. The new definition is able to better capture the syndrome, but studies have suggested further improvements can be made [118–120].

Following the AECC and Berlin definitions, chest imaging is often performed before a final diagnosis is made. Imaging can delay diagnosis and intervention as well as add subjectivity by interpreting imaging results [1]. In particular, to provide prompt recruitment and maintenance, a recruitment manoeuvre should be performed as soon as possible for any MV patient to open up the lung and assist with gas exchange [19, 20]. Final diagnosis should then follow patient stabilization.

Equally importantly, ARDS is typically diagnosed at one moment in time near the start of ventilation [121]. However, current MV settings, such as PEEP, at that time can greatly affect both the measured PaO_2_ and the required FiO_2_ for adequate oxygenation. Estenssoro et al. [121] reported that PEEP > 0 during initial ventilation can improve PaO_2_ so drastically that ARDS may be misdiagnosed if assessment is delayed too long, due to delay in blood gas extraction or waiting for chest imaging results. If each patient had been evaluated 6 h later, 52% would no longer fulfil the AECC definition for ARDS (P/F < 200) and instead be diagnosed with less severe ARDS or ALI [121], possibly changing their overall treatment and care. In this specific study, no RMs were performed, and Estenssoro et al. attributed the improvement in P/F ratio from < 200 to > 200 over 24 h for 18 of the 48 similarly ventilated patients to a relatively higher mean PEEP of 12.8 cmH_2_O after 24 h. Thus, initial care choices can interact significantly with the diagnosis made and thus, the subsequent care provided.

Villar et al. [122] conducted a similar trial to Estenssoro et al., where patients were tracked. Approximately 40% of ARDS patients exhibited an increase in P/F ratio to above the AECC threshold when evaluated after 24 h at PEEP ≥ 10 cmH_2_O and FiO_2_ ≥ 50%. Villar et al. [123] warns that all respiratory failure patients start off with poor oxygenation and neither the AECC or Berlin definitions allows for re-evaluation of hypoxemia at consistent ventilator settings, especially PEEP and FiO_2_. The authors recommend use of a PEEP–FiO_2_ trial conducted 24 h after ARDS diagnosis, stating this test would provide an easy and simple strategy to identify subpopulations and provide care based on actual patient risk [123].

Hence, defining and diagnosing ARDS/ALI within respiratory failure is an important step towards distinguishing between severe and moderate respiratory failure [118], and thus to providing appropriate care. However, the diagnosis of ARDS is slow and followed by rapid patient evolution. Care must thus be taken not to misdiagnose patients at the wrong time, or equally, to ensure that the diagnostic process does not lead to an inadequate level of care.

What is needed, is a consistent standard of care to provide early ventilation support for all patients requiring respiratory support so that patients could be diagnosed when convenient. Such an approach, if patient-specific, would allow early action in a consistent framework that does not currently exist in the field. These results thus also clearly show the need for patient-specific MV that can evolve as dynamically as the patient in the first 24–48 h.

### Summary

Mechanical ventilation management must carefully balance a diverse range of different ventilator settings. Insufficient or excessive support results in potential harm to patients, prolonging their dependency on MV. This issue is exacerbated by inter-patient variability in response across cohorts, as well as by intra-patient variability in the evolution of condition. There is thus a strong need for optimal titration mechanisms that are patient-specific, specific to disease state, and can evolve dynamically, in real-time in response to patient condition.

## MV strategies and clinical trials

There have been numerous ventilation strategies aimed at improving the quality of MV and patient outcomes [124]. Please refer to a recent review of the clinical advances for treatment of ARDS [1].

### Lung recruitment strategies: recruitment manoeuvres

Recruitment as defined by Fan et al. [113], is the “*dynamic process of reopening unstable airless alveoli through an intentional transient increase in transpulmonary pressure*”. Recruitment manoeuvres (RM) have shown to promote alveolar recruitment, increase end-expiratory volume, improve gas exchange, and attenuate VILI by preventing atelectrauma [113]. The tidal cycle during a RM shifts to where cyclic derecruitment is less likely to occur given that PEEP is greater than the closing pressure of the majority of alveoli. Early in the disease, atelectasis is reversible and the lung may be easily recruitable without negative side effects [125].

By transiently increasing pressure in the lung, collapsed or non-aerated alveoli have time to open and recruit. Alveolar recruitment increases the aerated lung volume aiding gas exchange and perfusion. The clinical benefits of RMs, particularly early in care, are numerous. However, the efficacy of RMs also degrade over time as the lung settles into a collapsed state and is thus again, more difficult to recruit [39, 126].

Evidence also suggests that high pressure RMs may overinflate parts of the heterogeneous ARDS or respiratory failure lung [127], and temporarily cause circulatory depression [128]. The links between respiratory failure severity, recruitability and other outcome measurements thus require further investigation and are not standardised, largely as a result of not being able to directly determine their impact on lung condition as they are conducted.

Rose et al. [3] described how effective RMs are difficult to conduct due to the heterogeneity of each patient and their response to increases of pressure. Specifically, for a given RM, some may over-distend and others fail to recruit [129], as lung elastance, the patient-specific response to pressure and volume varies. Thus, different types of RMs have been studied with inspiratory pressures anywhere between 30 and 60 cmH_2_O [19]. Sustained inflations or breath holding sessions for up to 40 s, intermittent sighs with high pressure or volume, and incremental increases in PEEP and/or PIP are also common types of RM. The goal in each case is to manage lung pressure to recruit more lung units without damaging others.

However, trials testing RMs have failed to produce consistent results and the best recruitment method is yet to be confirmed [113, 130]. Equally, some researchers have reported that not all patients benefit from an RM [17] or have any recruitable lung volume [121]. One recent meta-analysis [131] identified only 10 RCTs evaluating RMs and concluded that RMs decrease ICU mortality without increasing risk of barotrauma but found no effect on hospital or 28-day mortality. A second meta-analysis [132] reported a reduction in mortality pooling 6 RCTs as well as improved oxygenation and fewer rescue therapies. The authors of both meta-analyses noted the drastic differences between study designs and the prevalence of co-interventions (which together resemble the open lung approach) in the majority of the trials that may have confounded assessment of the effect of RMs. Thus, there remains a lack of confirmation of the long-term management, adverse effects, and generality of these findings in non-selected populations, further indicating a need for a patient-specific rather than cohort-specific, approach. Please refer to this recent review, and meta-analysis, of RMs [132].

### Setting MV using maximum compliance, inflection points

Static compliance has been reported to change significantly with both tidal volume and PEEP [133–136]. The local maxima of compliance at a patient-specific PEEP was noted to be dependent on the ventilated tidal volume and explained in terms of position on the static pressure–volume (PV) curve [133–136]. In general, higher tidal volumes reduce the PEEP of maximum compliance [133–136], and equally, higher PEEP at a fixed tidal volume can have a similar effect [105].

The gold-standard approach to obtain a static PV curve is the super syringe method that quasi-statically fills the lung before emptying it in a controlled stepwise manner allowing equilibrium in between each step. The points are connected to form the sigmoidal static PV curve. The produced loop can be used to optimise PEEP and tidal volume. Specifically, PEEP can be set in between the lower inflection point (LIP) and upper inflection point (UIP) of the static PV curve.

The super syringe method is clinically cumbersome and high workload for clinical staff, as it requires detachment from the ventilator [137, 138] for up to 15 min [24]. Static PV curves can be obtained directly from some modern ventilators, but require the patients to be sedated and thus are still a significant interruption to care [24]. Hence, these curves are not typically or regularly assessed for clinical use.

Stahl et al. [139] described the potential in monitoring dynamic respiratory mechanics over incremental PEEP, such as a staircase RM, to estimate both mechanics and recruitment simultaneously. They reported that dynamic respiratory mechanics could be used as a diagnostic tool and would be more appropriate than using static mechanics. Comparing static and dynamic compliance, Stahl et al. reported that dynamic compliance was less than static compliance, but the difference was dependent on alveolar pressure [139].

Overall, static compliance neglects airway resistance and misrepresents lung dynamics by assuming a static or quasi-static condition, when regular MV breathing is dynamic. Monitoring dynamic compliance results in smaller compliance (higher elastance) values, indicating the significance of dynamic effects. It is possible to track breath-to-breath dynamic compliance (or elastance) over incremental PEEP [140, 141]. Thus, tracking dynamic compliance or elastance as a surrogate of lung condition can be used to quantify recruitment and guide care [24].

Of note, the use of the term static compliance or dynamic compliance are interchanged between studies [142]. Definitions in medical texts [44, 57], may be different from other studies [129, 139, 143]. Thus, it is important to be clear on the concept and application of each definition and method, to avoid misinterpretation of these findings.

### Lung protective strategies

As a result of the difficult trade-offs between benefit and risk of MV, the goals of MV have changed over the last two decades. In particular, from specifying oxygenation goals, to a more cautious approach focusing on minimising VILI, while maintaining acceptable ventilation [101]. Avoiding atelectasis and overdistention of alveoli can attenuate alveolar and systematic inflammatory responses [22] and should translate into a measureable improvement in ARDS/ALI patient outcomes [2]. Hence, protection or risk mitigation has become a primary treatment endpoint.

A protective ventilation strategy is one aiming to minimise VILI and find a balance between oxygenation and CO_2_ elimination targets. Rose et al. [3] described the “*mortality reducing effect of lung protective ventilation using low tidal volumes and pressure limitation*” to prevent alveolar collapse or overdistention in ARDS patients. The study also suggested that these strategies might also be beneficial in patients with normal lungs. The following sections summarise several lung protective strategies examined.

#### The ARDS network trial

The ARDSNet strategy aims to minimise distension-induced lung injury, while maintaining acceptable oxygenation, by ventilating with small tidal volumes (≤ 6 mL/kg) and plateau pressures lower than 30 cmH_2_O [26]. The ARDSNet trial showed that lower tidal volumes (6.2 ± 0.8 mL/kg) are better than higher (11.8 ± 0.8 mL/kg). This low V_t_ strategy uses tables of fixed combinations of FiO_2_ and PEEP that are periodically adjusted to maintain oxygenation goals [26]. ARDSNet is easy to follow, but relies on the relationship between PaO_2_ and FiO_2_ being generic to all patients at all times as their condition evolves.

However, the physiological rationale and the ‘one size fits all’ lack of individuality in care has been questioned [26]. The ARDSNet protocol may also be associated with increased atelectasis due to the low PEEP used and lack of RMs [26]. Thus, to avoid VILI by minimising lung strain and further improving care, the ideal tidal volume should be monitored in a patient-specific breath-by-breath or high time resolution approach similar to PEEP [24].

#### The open lung approach

The open lung approach (OLA) aims to open and maintain lung recruitment [1]. It also reduces dynamic strain [26]. RMs are used to open up the lung and PEEP is titrated to gas exchange or respiratory variables to maintain recruitment, avoiding cyclic collapse/re-opening [26].

##### Cohort based OLA approaches using PEEP–FiO_2_ tables or oxygen saturation goals

Three significant, early clinical trials of OLA strategies are ALVEOLI [8], LOVS [10], and EXPRESS [11]. ALVEOLI used two predetermined PEEP–FiO_2_ tables to ensure SpO_2_ was within acceptable limits of 88–95% [8]. The lower PEEP group represented clinical consensus in 1995, whereas the higher PEEP group reflected the beneficial results of Amato et al. [7]. ALVEOLI used a target V_t_ of 6 mL/kg predicted body weight with PIP limited to 30 cmH_2_O or less. LOVS [10] also utilised PEEP–FiO_2_ tables with V_t_ of 6 mL/kg, but conducted a 40 s breath-hold at 40 cmH_2_O with FiO_2_ of 1.0 and thus allowed PIP up to 40 cmH_2_O. EXPRESS [11] took a different approach by adjusting FiO_2_ to maintain oxygenation goals for the participants in the ‘minimal distension’ control group. PEEP and PIP were kept as low as possible without dropping out of an acceptable oxygenation range. Within the increased recruitment group, PEEP was kept as high as possible preventing PIP rising above 30 cmH_2_O regardless of the effect on oxygenation.

None of these three large multi-centre trials conclusively reported any benefit of the OLA or higher PEEP ventilation. However, EXPRESS did report a significant improvement in ventilator free-days (median [IQR]: 7 [0–19] vs 3 [0–17], p = 0.04). Although the primary outcome of mortality failed to reach significance, benefits including higher compliance values, improved oxygenation [8, 11], and reduced rates of, and death from, refractory hypoxemia [10], were achieved.

##### OLA approaches using PEEP based on static pressure–volume curves

An early, small trial by Amato et al. [7] reported a benefit from higher PEEP ventilation above the LIP on a PV curve compared to standard ventilation. Mortality was reduced from 71 to 38% and rates of barotrauma were greatly reduced (n = 53, p < 0.001). Villar et al. [9] conducted a similar trial, setting PEEP 2 cmH_2_O higher than the LIP and reported significant improvements in ICU and hospital mortality, ventilator free days, and organ failures (n = 95). However, Oba et al. [12] argued that the static pressure–volume curve may not be the best way to select optimal PEEP for an OLA.

##### OLA approaches using PEEP based on dynamic compliance/elastance

Spieth et al. [26] conducted an animal trial involving pigs with surfactant washout induced ARDS, randomised into either the standard ARDSNet protocol (PEEP = 12 cmH_2_O) or an open lung approach (OLA) with PEEP set to minimal respiratory elastance. PEEP and mean airway pressure were higher in the OLA. OLA was associated with improved oxygenation after 6 h and redistributed pulmonary perfusion, but with more alveolar overdistension, while ARDSNet was associated with more intra-alveolar haemorrhage. Inflammatory mediators and markers of lung parenchymal stress did not differ significantly. Better redistribution of pulmonary blood flow in the OLA approach may contribute to better ventilation-perfusion matching and the reported improved oxygenation.

Another, more recent, animal study by Santos et al. [144] compared the pulmonary vascular mechanics of three groups each with PEEP set based on the maximal compliance PEEP, (1) hyperinflation = 6 cmH_2_O above, (2) OLA = 2 cmH_2_O above, and (3) collapse = 6 cmH_2_O below. The OLA group displayed the lowest pulmonary artery resistance, effective arterial elastance, and reflection coefficient. The authors concluded that OLA was the best setting. Therefore, a patient-specific OLA strategy providing PEEP corresponding to minimal elastance (or maximal compliance) is beneficial in pigs, but remains untested against clinical outcomes in a randomized controlled trial in humans.

There are two recent studies that have shown promising results of the OLA. First, a multicenter RCT, by the Open Lung Approach Network [145], screen 1874 patients under ‘standardized ventilator settings’ (FiO_2_ ≥ 0.5, PEEP ≥ 10 cmH_2_O) and included patients with ARDS (P/F ratio ≤ 200 mmHg) into either a control ARDSNet group (n = 101, PEEP set by the ARDSNet protocol [5]) or a OLA group (n = 99, PEEP = PEEP at maximal dynamic compliance). Both airway driving pressure and P/F ratio improved significantly at 24, 48, and 72 h for the OLA group as compared to ARDSNet group. Although the study was statistically underpowered to significantly show reductions in mortality, a non-significant trend of lower mortality was observed (60 day: 29% vs 33%, p = 0.18; ICU 25% vs 30%, p = 0.53). Ventilator-free days were similar in both groups (8 [0–20] vs 7 [0–20], p = 0.53). The Open Lung Approach Network concluded that OLA improves oxygenation and driving pressure without detrimental effects, which supports a larger multicenter OLA trial. The careful design of performing patient screening under standardized conditions ensures that inclusion is not biased by the current ventilator settings that are known to affect P/F ratio and subsequent ARDS diagnosis [121, 122].

The second study also examined the effect of OLA but instead in a cohort of major abdominal surgery patients [146]. Patients were assessed after a 30-min period of standardized ventilation (V_t_ = 6 mL/kg, PEEP = 5 cmH_2_O) and subjected to a RM after which each patient was randomized into either a control group (n = 18, PEEP = 5 cmH_2_O), or an individualized open-lung PEEP group (n = 18, PEEP = PEEP at maximal dynamic compliance). Compared to the condition prior to the RM, the OLA group displayed an improvement in both: (1) an increase in compliance and (2) a decrease in driving pressure, whereas the control group did not. Despite being performed in anesthetized healthy individuals, opposed to ARDS patients, this study further validates the intuition behind the OLA, RMs and individualized PEEP settings.

Overall, different studies of OLA strategies have shown modest clinical benefits. However, improvements in primary patient outcomes have not yet been fully established by large multi-centre trials [7–11, 145]. Again, the lack of a patient-specific approach is a potential limiting factor that could impede some of these studies. The more recent OLA results [145–147] point towards a patient-specific compliance/elastance as a means of obtaining the best benefits of an OLA approach.

### Variable ventilation strategies

Healthy physiological systems exhibit a natural variability that leads to greater flexibility and more robust function compared to diseased systems. In contrast, a low variability breathing pattern may be observed in MV patients who failed to wean from MV. Thus, it has been proposed to reintroduce variability to replicate this behaviour [148].

Variable controlled ventilation has been associated with improved oxygenation, a reduction in mean peak airway pressure, as well as improved pulmonary function in several animal studies [149–153]. The best results occur when tidal volume variability matches the variability in healthy subjects [154]. The mechanisms responsible may include recruitment, surfactant release and improved volume/flow matching because of the redistribution of pulmonary blood flow [151, 152]. It is hypothesized that variable tidal volumes improve pulmonary function by replicating natural variability.

Neurally adjusted ventilation assist mode (NAVA) [155], is an assist ventilation mode that uses patient diaphragm electrical activity, *Eadi*, to trigger and cycle off the ventilator support. It also uniquely delivers airway pressure in proportion to this measured *Eadi*, matching ventilator support with patient’s demand. NAVA was found to improve patient-ventilator interaction, reducing the number of asynchrony events [156], as well as increasing respiratory variability in *Vt* and flow related variables [157]. Studies on NAVA have reported on impact to the patients [158–160], and an optimal titration method [161, 162]. Thus, the benefits of this approach are potentially more substantial for patients ventilated for long durations. However, similar to other ventilation strategies, there is also limited guideline is setting the appropriate NAVA level [161–163] of pressure support, so its use is affected by this difficulty and its added cost.

### High frequency oscillatory ventilation (HFOV) and airway pressure release ventilation (APRV)

In both these approaches, a relatively high mean airway pressure, referred to as ‘continuous distending pressure’ in HFOV or ‘P_high_’ in APRV, is used to maintain a healthy to high end-expiratory lung volume and adequate oxygenation levels. Both methods promote alveolar recruitment and maintenance due to the continuously elevated pressure within the lung, and minimising atelectasis as the minimum pressure is relatively high. Recruitment not only depends on the pressure in the lung, but also the duration that pressure is held, where elevated pressures for a relatively long time can assist with opening more stubborn, stiffer alveoli.

HFOV ventilation uses rapid application of small tidal volume breaths with a large mean airway pressure to ensure adequate oxygenation [101, 164, 165]. However, due to the small tidal volume breaths, the patient’s lung has a large proportion of dead space and reduced alveolar minute ventilation with less CO_2_ clearance. The majority of HFOV studies treated neonatal patients and reported small reductions in chronic lung disease [101]. Recent studies to compare HFOV with conventional MV have reported promising physiological and inflammatory results [101, 165] but others have reported no effect on patient outcomes [166]. Moreover, recent guidelines [28] considering six RCTs strongly recommend that HFOV should not be routinely used in moderate-severe ARDS patients. The deleterious effects of HFOV, observed in some studies, may be attributed to higher airway pressures causing negative hemodynamic consequences [1].

APRV is similar to HFOV, but includes brief periods where the pressure is dropped to release air from the lungs and eliminate CO_2_. These pressure releases must be kept brief to prevent the pressure dropping to a point where the alveoli may start to collapse. The pressure releases are crucial to eliminate respired CO_2_ out of the lung. Thus, patients that have hypercapnia should be ventilated with more frequent or longer duration releases, whereas patients that have hypoxemia require fewer and shorter releases requiring patient-specific adjustment [167]. The balancing point has yet to be determined in groups where both hypoxemia and hypercapnia occur together. APRV facilitates spontaneous breathing and has been associated with progressive recruitment [168], improved oxygenation [52], reduced peak airway pressures [52, 168], and improved volume/flow matching [169] however, a recent review [170] described ‘tremendous variation’ in published settings referred to as APRV limiting definitive conclusions and clinical application.

Although HFOV and APRV are protective strategies operating on similar principles, their applications are quite different. HFOV requires sedation and has not been conclusively proven in adult human trials [1, 28]. APRV on the other hand, has been shown to be clinically beneficial in spontaneously breathing patients, but is still relatively new in practice. Neither is necessarily patient-specific in its application, thus still relying on clinical judgement to find optimal settings, and neither has reached a state of regular use or wide uptake.

### Summary

In summary, it is currently clear that no general, “*one size fits all*” cohort-specific protocol is broadly successful. It is equally clear that respiratory failure patients requiring mechanical ventilation are highly variable and there is a need to manage mechanical ventilation strategy based on patient-specific needs, rather than per cohort. Hence, it is very important to have the means to assess the dynamic changes in patient-specific respiratory disease state regularly in clinical real-time without additional invasive measurements or interruptions to care. Different strategies, each based upon different intuition on how ventilation can be improved, have been presented. To summarize their commonalities and differences, Table 1 briefly summarizes the aims of recent research, the driving intuition behind how each strategy intends to address the various problems facing MV in clinical practice and the limitations of the strategy. One trend apparent in Table 1 is that many recent research areas focus on the related problem of inter- and intra-patient variability with the intention that personalized ventilation will avoid VILI and enable application to any patient requiring MV without diagnosis of ARDS.

**Table 1:** MV strategies previously, or currently, studied to address the major problems facing clinical utilization of MV. The intuition behind the strategy, the aims of recent studies, and the limitation of current methods are briefly summarized

	Study aims	Intuition	Limitation
Recruitment manoeuvres			
Stepwise recruitment, maximum recruitment	Research the role, safety, clinical feasibility, and adverse effects of single, and/or regular, recruitment manoeuvres	Recruiting lung volume early improves ventilation and prevents atelectrauma but excessive pressures may further injure the lung	Each patient will respond differently to recruitment depending on the condition of their lung, the RM procedure could be routine but the ventilation settings determined afterwards should be specific to the patient at that moment in time
Compliance/elastance			
Setting MV using maximum or inflection compliance	Employ clinical protocols to determine an optimal ventilation PEEP using a patient’s static compliance/elastance and inflection	The best way to model a patient’s lung condition is to measure its compliance in a static PV curve	However, doing so is invasive and an impediment to continuing ventilation and is not feasible for frequent reassessment
Dynamic monitoring	Employ clinical protocols to determine an optimal ventilation PEEP using a patient’s dynamic compliance/elastance and inflection and often mathematical methods	Patient airway dynamics are going to change overtime (e.g. pre- and post-recruitment or PEEP change), modelling compliance/elastance from pressure/volume data can enable incremental improvements to ventilation settings without large digressions from ventilation	Reliance on mathematical models may cause adverse effects to be ignored. Moreover, to ensure the current setting remains optimal, small perturbations are necessary which may disrupt ventilation
Lung protective strategy			
ARDSNet, OLA, EXPRESS	Employ clinical protocols that can be used to select ventilation parameters all within acceptable ranges to prevent further lung injury	To prevent further lung injury, ventilation should be set within canonically safe ranges of tidal volumes, plateau pressure, driving pressures, PEEP etc	Unfortunately, respiratory failure patients are diverse and what may be safe for the majority may be detrimental for some
Variable ventilation			
NAVA	Improve patient-ventilator interaction by promoting patient spontaneous breathing	Healthy breathing is variable over time and without this variability a patient’s breathing efforts may be suppressed. To promote breathing effort, variable breaths are delivered either artificially or using the electrical activity of the diaphragm	Each patient may respond differently to variation and relatively little comprehensive protocols or guidelines exist
High mean pressure modes			
HFOV, APRV	Development of clinical protocols to prevent atelectasis with continually high airway pressures	To prevent collapse or atelectasis, continually high airway pressures are used which result in a healthy to high end-expiratory lung volume	Neither HFOV nor APRV are patient-specific. Moreover, the small tidal volumes at high pressures create dead space and reduce minute ventilation and CO_2_ clearance over alternatives

## The path forward for mechanical ventilation and the role of engineers

Mechanical ventilation is straightforward in principle. However, it is complex in implementation, due partly to heterogeneity of disease and partly to the individual’s response to treatment. There thus remains a need to improve MV management and engineers can play an important role in both research and development of those next-generation solutions.

### Managing inter- and intra-patient variability

Due to inter- and intra-patient heterogeneity, ventilating respiratory failure patients with generalised approaches will not cater for patient-specific needs. What is needed, is the ability to accurately assess lung condition non-invasively and in real-time with no interruption to ventilation. In addition, the lung condition of an ARDS or respiratory failure patient can change dramatically over the first 24 h of ventilation [171, 172].

In particular, lung condition improves with effective MV, which in turn, can significantly alter the patient’s need for subsequent ventilation [60]. Thus, instead of selecting PEEP once at the start of MV [7, 9], or only occasionally, ventilation settings should be constantly titrated, either breath-to-breath or very regularly. These changes should occur in clinical real-time to evolve with the patient and reflect their current condition, preferably in an automated fashion that is not burdensome to care givers [173]. However, this outcome requires automated data acquisition, and computational modelling and methods, to provide to necessary monitoring and decision support. More specifically, automated ventilation strategies that cater for individual, patient-specific needs can potentially be realised with the advancement of engineering technologies and model-based methods.

### Improving technologies and model-based methods

#### Lung imaging technologies

Lung imaging is one means of providing regular monitoring of lung condition. Various imaging technologies have emerged to help clinicians diagnose patient condition. These technologies, such as EIT [174], ultrasound [175–177], or low dose CT [178], provide potential means to measure lung condition. These imaging techniques are comparatively non-invasive, and can be applied frequently in MV patients to potentially improve MV delivery. However, there is significant need for improvement.

For example, EIT operates regionally, capturing lung images representing a 5–10 cm cross-section relative to its position [70]. Thus, application of EIT is limited to regional lung recruitment monitoring and extrapolation to global measures of ventilation. Expanding EIT to multi-slice or complete lung imaging would require a significant increase in the number of required sensors, and thus the required computing resource for signal processing and image reconstruction, all of which may limit real-time analyses. Significant research is required to optimise the architecture and implementation of a real-time, complete lung EIT system, from computational methods to speed solution to more advanced and accurate inverse models.

In addition, EIT, CT or ultrasound images do not provide absolute and objective measurement of lung condition. Specialised clinicians are required to interpret images, diagnose and recommend treatment. In addition, differences in clinician background and experience may potentially lead to inconsistent evaluation, yielding errors in decision-making and treatment recommendations that may harm patients. Further research is required in these existing imaging technologies, including improving image quality, and providing more objective information that does not rely on clinical experience and evaluation for robust clinical decision-making which, if successful, will ultimately improve patient care.

#### Research in novel ventilation modes

New ventilation modes such as NAVA [179], variable pressure support [151], automatic protective ventilation [173], proportional assist ventilation [180] and other non-conventional techniques [181] were developed as means to improve MV treatment. Unfortunately, the application of these MV modes remains limited due to a lack of clinical guidelines, based, in turn, on lack of methods to monitor and optimise their use for specific patients who might benefit. In addition, there is currently a lack of evidence, supported by clinical studies with sufficiently large patient cohorts, to justify application of these modern MV modes into daily clinical practice, in turn limiting the data available to develop the engineering tools and methods to optimise their use.

Fortunately, these ventilation modes are becoming increasingly popular in engineering signal processing research. In particular, clinically useful decision support systems are being developed to help clinicians interpret patient condition, as well as guide clinicians to better manage MV. For example, Moorhead et al. studied potential models and metrics in setting NAVA level [157], Sinderby et al. have developed a neural index to quantify the quality of patient-ventilation interaction during NAVA [182], and Pomprapa et al. have investigated lung protective ventilation with the use of EIT [183]. Engineering research was crucial for the development of these MV modes and will continue to be critical to their validation and implementation.

#### Model-based methods

One prospective topic of MV engineering research relates to applying model-based algorithms in conjunction with specialised clinical protocols. Mathematical modelling of the respiratory system, and its effects on the cardiovascular system, allow estimates of a patient’s response to changes in their treatment, which can allow virtual trials [184] or provide recommendations to a physician as part of a clinical decision support system [185].

##### Mathematical modelling

Model-based research can include development of mathematical models and validating model relevance, as well as identification of parameters, conducting decision metric studies, and clinical trial development. All these outcomes also lead toward validating the efficacy and generalizability of physiological models at the bedside.

One popular model-based research area is mathematical modelling of lung physiology. These models, ranging from simple to complex forms [186–190], describe lung physiology to help researchers better understand lung conditions. Some models can also be used to assess real-time patient-specific condition [25, 105, 191–193], recruitment status [24, 191, 193, 194], and patient-specific response to MV [191, 193, 195]. Importantly, they all offer insight into patient-specific condition that is not available via typical static surrogate estimates [189, 196], and, they can be estimated breath-to-breath, and monitored as a surrogate of patient condition without interruption. As a result, they have the potential to be applied in MV management [105, 191, 193, 197]. Reviews of lung modelling exist [198, 199] but unfortunately, few focus on clinical utility, and none, that we are aware of, outside our group [24].

##### Validation of model-based methods

Mathematical models are typically developed and validated with data derived from animal or human studies. An extension of these ‘*in*-*silico*’ simulations is the concept of virtual patients: a dataset of patients, including both ventilation and physiologic data, that can be used to test and validate mathematical models and their utility to measure disease and provide recommendations. A recent review on model-based therapeutics appeared, from our group in this journal, which covered modelling of virtual patients and their validation, which is a major step towards using models in clinical application to guide care [184].

The natural next step beyond simulation, or virtual patient, based studies are prospective studies where current ventilation settings and physiologic measurements are used in a feedback loop to influence further MV therapy. Several groups have developed methods to provide ‘automatic’ MV, using physiologic measurements with standard protocols or closed-loop feedback systems, to fine-tune ventilator settings during MV however, these works are currently limited to animal studies [183, 200]. Similarly, non-model-based studies have assessed the feasibility of recommending optimal PEEP in small studies using EIT for example [201]. These studies have shown the feasibility of closing the feedback loop to provide near autonomous MV within prescribed parameters.

Personalizing MV to account for inter- and intra-patient variability using mathematical modelling has been described in simulations [185], retrospective comparison studies [202] or prospective feasibility/pilot studies aiming to provide recommendations [105, 203, 204]. One particular group has recently commercialized their physiological modelling research into a decision support system however, their published results are also limited to small, feasibility studies [205, 206]. To our knowledge, no large, outcome-focused trial assessing the clinical utility and efficacy of model-based MV has been published. Thus, despite the potential of model-based methods to guide care, as seen in other areas [184], there are yet to be any clinical trials comparing outcomes of a model-based MV intervention, against standard practice, barring the ongoing CURE^1^ trial [207], which is recruiting slowly at this time, and the recently initiated CARE^2^ trial in Malaysia (unpublished).

##### Next steps in model-based MV

Implementation of any automatic or prescriptive MV system, model-based or otherwise, must consider the ramifications of MV in a patient-specific manner. Some ventilated patients develop asynchrony, expiratory flow limitation or negative hemodynamic consequences during MV. Asynchronous (or dyssynchronous) breathing occurs when the MV is mis-matched with the patient’s breathing efforts. Improved signal processing may improve robust application of model-based methods [208]. Moreover, improved modelling, for example adding EIT signals, may improve alignment between the ventilator and the patient by modelling patient efforts. Expiratory flow limitation (EFL) is believed to occur when smaller airways constrict during expiration, isolating areas of residual high pressure and thus limiting gas exchange [209]. Appropriate MV settings, especially PEEP, for patients with EFL can be very different than typical care; improved modelling could aid detection and PEEP titration to deliver appropriate patient-specific care [209]. MV intended to be protective can negatively affect hemodynamics in ARDS patients [1]. Improved modelling of the cardiopulmonary system could yield insights into the hemodynamic effects of MV and mitigate the consequences that have lead to early stopping of some clinical trials. Biomedical engineers have a valuable opportunity to advance clinical practice by combining clinical study results and intuition with individualized modelling of a complex, interrelated biomedical system.

Overall, mathematical models can generate insight into pulmonary physiology and patient-specific ventilation and perfusion in response to ventilator settings. Equally, forecasting poor responses can prevent negative clinical outcomes, such as desaturation or lung collapse and thus further improve patient care at the individual level. There is thus an emerging capability for clinicians to titrate patient-specific care using such computational models developed in collaboration with engineers. Coupled with regular recruitment and other protective lung strategies, model-based treatment offers the opportunity for clinicians to provide patient-specific therapy in a consistent fashion. In essence, software services augment the ventilator hardware and ventilator modes to further monitor and optimise care.

Finally, model-based MV research is not without challenge. It is often hindered by the need of specialised clinical protocols [38, 210–213], results comparison and validation [62, 214, 215], data quality, and model identification [190, 212, 216]. Ultimately, the need for large randomised controlled trials is a further limitation. As a result, to date, models have not yet been used to prospectively guide therapy directly in a larger setting [105, 197], although pilot trials have been conducted [25, 207, 217]. Hence, the need of engineering methods to improve mechanical ventilation management is imminent and it is paramount for engineers to understand clinical aspects of mechanical ventilation and potential advancement.

## Conclusion

The overall review summarises the clinical state of the art in the research and application of MV in an engineering context. In general, existing MV management is general and describes a ‘*one size fits all*’ cohort-based approach that does not address the heterogeneity of the ventilated patient lung, nor the inter- and intra-patient variability critically ill patients exhibit. It is thus not able to deliver further improvements in clinical outcomes without a drastic change towards a patient-specific approach that exploits data captured non-invasively in real-time.

New technologies and model-based ventilation have been gaining ground in MV research. Model-based estimation enables the clinician to evaluate, otherwise unavailable, patient-specific lung condition, monitor patient evolution, and, once informed, select an optimal MV setting guided by a clinical protocol. Such a development provides an individualised, patient-specific or personalised ‘one *method* fits all’ approach that monitors every breath and guides therapy in real-time, including recruitment manoeuvre interventions and timing. This model-based approach in particular provides a means for engineers and clinicians to collaborate to create personalised next-generation solutions to this significant and costly health care problem, thus improving clinical ability to provide safe, effective MV beyond what is possible today.

## Abbreviations

AECC: American-European Consensus Conference
ALI: acute lung injury
APACHE: acute physiology and chronic health evaluation score
APRV: airway pressure release ventilation
ARDS: acute respiratory distress syndrome
CT: computed tomography
EIT: electrical impedance tomography
EFL: expiratory flow limitation
FiO_2_: fraction of inspired oxygen
HFOV: high frequency oscillatory ventilation
ICU: intensive care unit
I:E: inspiration to expiration
LoMV: length of mechanical ventilation
LIP: lower inflection point
MV: mechanical ventilation
NAVA: neurally adjusted ventilation assist
NIV: non-invasive ventilation
OLA: open lung approach
P/F: PaO_2_/FiO_2_
PaO_2_: partial pressure of oxygen
*P*_aw_: airway pressure
PEEP: positive end expiratory pressure
PIP: peak inspiratory pressure
*P*_plat_: plateau pressure
PV: pressure–volume
RM: recruitment manoeuvre
RR: respiratory rate
SAPS: simplified acute physiology score
SIMV: synchronized intermittent mandatory ventilation
SOFA: sequential organ failure assessment score
SpO_2_: peripheral capillary oxygen saturation
UIP: upper inflection point
VFD: ventilation free days
VILI: ventilator/ventilation induced lung injury
*V*_*t*_: tidal volume

## Footnotes

1 https://www.anzctr.org.au/Trial/Registration/TrialReview.aspx?id=366838.

2 https://www.anzctr.org.au/Trial/Registration/TrialReview.aspx?id=373157.
